# The Antibacterial Activity of Kaempferol Combined with Colistin against Colistin-Resistant Gram-Negative Bacteria

**DOI:** 10.1128/spectrum.02265-22

**Published:** 2022-10-31

**Authors:** Huijing Zhou, Mengxin Xu, Wenhui Guo, Zhuocheng Yao, Xin Du, Lijiang Chen, Yao Sun, Shiyi Shi, Jianming Cao, Tieli Zhou

**Affiliations:** a Department of Clinical Laboratory, Key Laboratory of Clinical Laboratory Diagnosis and Translational Research of Zhejiang Province, The First Affiliated Hospital of Wenzhou Medical University, Wenzhou, China; b Department of Medical Laboratory Science, School of Laboratory Medicine and Life Science, Wenzhou Medical University, Wenzhou, Zhejiang Province, People's Republic of China; Lerner Research Institute

**Keywords:** colistin-resistance, Gram-negative bacteria, biofilm, kaempferol, synergistic effect

## Abstract

Social hygiene is seriously threatened by the rise in colistin (COL) resistance against Gram-negative bacteria (GNB). With resistance to last-line antibiotics such as COL becoming more common, it is imperative to identify alternative treatment options. In our work, we sought to determine if COL plus kaempferol (KP) present synergistic effects on the antibacterial and antibiofilm activities against colistin-resistant (Col-R) GNB *in vivo* and *in vitro*. Twenty-four Col-R GNB were collected as the experimental strains. The synergistic activity of COL and KP was evaluated by checkerboard method, time-killing assays, and the Galleria mellonella experiment. The antibiofilm effectiveness of the COL/KP combination against Col-R GNB was assessed using biofilm inhibition and eradication assays and scanning electron microscopy (SEM). Cytotoxicity tests were performed to detect the toxicity of KP monotherapy or combination therapy. There is synergistic antibacterial activity of COL and KP combination *in vitro*. KP combined with COL could inhibit the formation of bacterial biofilms. The amalgamation of COL and KP considerably reduced the amount of bacteria in the biofilm, according to the SEM findings. The COL/KP combination improved the survivorship of infected larvae in the G. mellonella *in vivo* infection model. In addition, the combination of KP and COL showed no cytotoxicity at synergistic combined concentrations according to cytotoxicity assays. This represents the first account of the antibacterial and antibiofilm activities of KP in combination with COL against Col-R GNB. Therefore, our results may provide an effective alternative route to combat Col-R GNB infections.

**IMPORTANCE** COL is one of the few antibiotics effective against clinical isolates of GNB. However, in recent years, GNB resistance to colistin has been increasing. As a result, the combined application of colistin in conjunction with nonantibacterial medications has garnered considerable interest. In this work, the KP/COL combination showed effective antibacterial and antibiofilm activities *in vitro* and *in vivo*. The synergistic effect of combined application may be attributed to membrane permeability. Due to the low cytotoxicity of the combined concentration, the combination exhibits a promising future for use in clinical anti-infection treatments. This finding might broaden the potential applications for COL.

## INTRODUCTION

Globally, multidrug-resistant Gram-negative bacteria (MDR-GNB) pose serious health risks (1). All of these have led to the re-evaluation of outdated medications like polymyxins that were deemed to be too toxic for clinical usage (1). In the mid-1990s, colistin (COL) re-emerged as a last-resort therapy for MDR-GNB. Unfortunately, with COL's comeback, there is now widespread opposition against it (1). New methods are urgently required in this situation to increase the efficacy of COL or to stop the rise of infections that are resistant to it. The preclinical stage of the discovery of new antibiotics is not only slow and difficult, but also carries a significant chance of failure (2). Along with creating innovative antibiotics, the combination of traditional antibiotics and nonantibacterial drugs is also a rapid and effective treatment option.

About 80% of bacterial infections are caused by bacterial biofilms, which are the most resilient type of bacterial aggregation (3). Biofilm is a complex, sessile population of bacteria that can be found adhering to a surface or firmly lodged as aggregates in an extracellular matrix (4). Bacteria are resistant to antibacterial treatments because of the biofilm matrix that surrounds them (4). Additionally, because the antibiotics currently on the market have higher minimum inhibitory concentrations (MICs) and minimum bactericidal concentrations (MBC), which could cause *in vivo* toxicity, they are ineffective for treating these biofilm-related infections (4). No specific medications that target bacterial biofilms are currently being used in clinical trials (5). Therefore, it is crucial to create antibiofilm treatments that can efficiently reduce and get rid of illnesses caused by biofilms.

A flavonoid called kaempferol (KP) is present in many plants that are edible and in plants or botanical preparations that are frequently utilized in traditional medicine. KP and several of its glycosides exhibit a wide range of pharmacological actions, including anti-inflammatory, antioxidant, anticancer properties, and antibacterial, as demonstrated by numerous preclinical investigations (6). However, there are few studies on the antibacterial activity of KP. It is reported that KP demonstrated antibacterial activities against Propionibacterium acnes (7). Furthermore, Falcão-Silva et al. reported that kaempferol-3-O-beta-d-(6′'-E-p-coumaroyl) in combination with antibiotics, glucopyranoside (tiliroside), which was isolated from Herissantia tiubae (Malvaceae), reduced the MICs for norfloxacin (16-fold), ciprofloxacin (16-fold), lomefloxacin (4-fold), and ofloxacin (2-fold), as well as an impressive reduction in the MICs for the biocides (up to 128-fold) (8). Nevertheless, studies on the synergistic effects of KP plus COL in the management of Col-R GNB are currently lacking. Herein, the synergistic effect and antibiofilm of COL/KP combination on Col-R GNB clinical isolates were studied in order to offer fresh potential therapeutic approaches against resistance to this last line of antibiotics in the future.

## RESULTS

### Antimicrobial susceptibility testing.

The MICs of KP against all the 24 strains of Col-R GNB were ≥512 μg/mL, indicating that KP had no or low antibacterial activity against Col-R GNB. All strains were insensitive (intermediate or resistant) to COL, and the MICs of COL ranged from 4 μg/mL to >128 μg/mL (Table 1).

**TABLE 1:** Summary of MIC values and FICIs of colistin combined with kaempferol against the 24 colistin-resistant GNB clinical isolates

Species	Strains	Monotherapy (μg/mL)		Combination (μg/mL)		FICI	Potentiation^*a*^	Interpretation
		Colistin	Kaempferol	Colistin	Kaempferol			
P. aeruginosa	TL1671	128	512	8	8	0.078	16-fold	Synergistic
	TL1744	64	>512	2	8	0.039	32-fold	Synergistic
	TL2314	32	512	8	8	0.266	4-fold	Synergistic
	TL2902	128	512	2	8	0.031	64-fold	Synergistic
	TL3008	>128	512	16	16	0.188	>8-fold	Synergistic
	TL3086	>128	512	16	16	0.188	>8-fold	Synergistic
E. coli	DC3599	8	>512	2	8	0.258	4-fold	Synergistic
	DC3806	8	>512	2	128	0.375	4-fold	Synergistic
	DC3846	4	>512	0.5	16	0.141	8-fold	Synergistic
	DC4887	16	>512	8	0.5	0.501	2-fold	Additive
	DC5262	>128	>512	0.5	16	0.018	>256-fold	Synergistic
	DC7333	8	>512	1	8	0.133	8-fold	Synergistic
K. pneumoniae	FK1913	>128	>512	1	8	0.012	>128-fold	Synergistic
	FK3810	8	>512	2	512	0.75	4-fold	Additive
	FK3994	128	>512	32	16	0.266	4-fold	Synergistic
	FK6556	16	>512	4	256	0.5	4-fold	Additive
	FK6663	128	>512	2	32	0.047	64-fold	Synergistic
	FK6696	64	>512	2	16	0.047	32-fold	Synergistic
A. baumannii	BM1539	8	>512	2	8	0.258	4-fold	Synergistic
	BM1579	16	>512	4	4	0.254	4-fold	Synergistic
	BM2370	4	512	1	8	0.266	4-fold	Synergistic
	BM2412	32	512	1	16	0.125	32-fold	Synergistic
	BM2431	4	512	1	8	0.266	4-fold	Synergistic
	BM2622	32	512	0.5	256	0.516	64-fold	Additive

a Multiples of MIC reduction compared with colistin alone.

### Synergistic antibacterial activity of kaempferol combined with colistin *in vitro*.

Synergistic actions were investigated using checkerboard assays. According to checkerboard tests, the MICs of COL were significantly decreased after KP was added. The results showed that 83% and 17% experiment strains exhibited synergistic and additive effects, respectively. None of the strains demonstrated an antagonistic effect. The COL/KP combination had a strong synergistic effect on most of the Col-R strains, with fractional inhibitory concentration index (FICI) ranging from 0.012 to 0.375 (Table 1).

### Antibiofilm activity of colistin combined with kaempferol.

**FIG 1:**
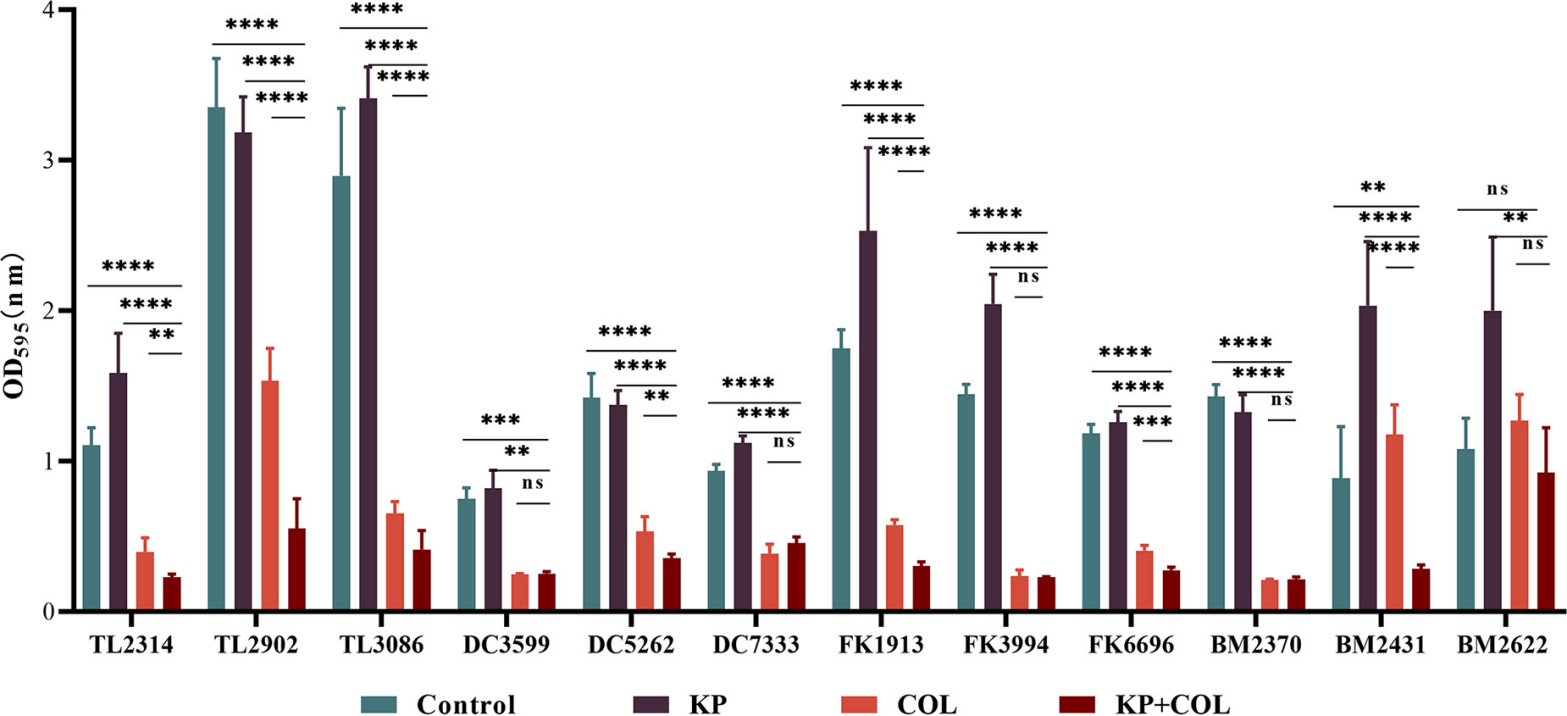
Biofilm inhibitory effects of COL combined with KP on Col-R GNB. Data were analyzed by Student's *t* test; (ns, not statistically significant; **, *P* < 0.005; ***, *P* < 0.001; ****, *P* < 0.0001). OD_595_, optical density at 595 nm. All data are reported as the mean ± SD (*n* = 3 per group) from two independent trials.

**FIG 2:**
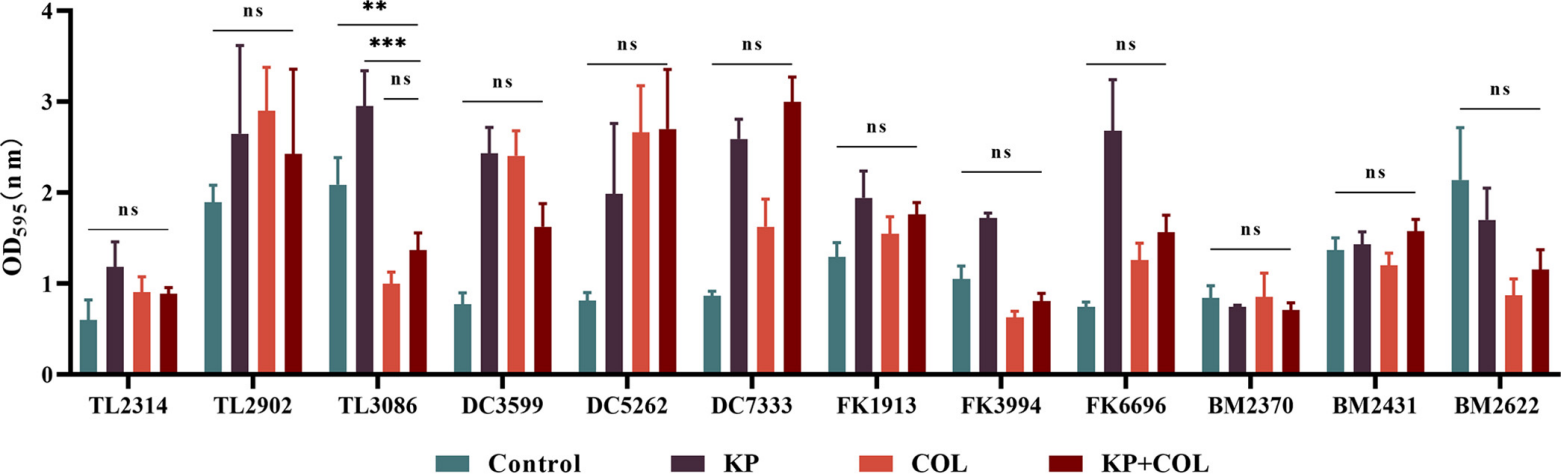
Biofilm eradication effects of colistin combined with KP on Col-R GNB. Data were analyzed by Student's *t* test; (ns, not statistically significant; **, *P* < 0.005; ***, *P* < 0.001). OD_595_, optical density at 595 nm. All data are reported as the mean ± SD (*n* = 3 per group) from two independent trials.

The capacity of KP and COL to prevent the growth of biofilms and destroy Col-R GNB strains' premade mature biofilms was examined using the crystal violet method. As shown in Fig. 1, compared with COL or KP alone group and control group, COL combined with KP (*P* < 0.05) could effectively inhibit biofilm formation in more than half of the Col-R strains. However, as shown in Fig. 2, the combining of COL and KP seemed to have no discernible removal impact on the developed biofilms of any of the tested strains compared to the control group (*P* > 0.05).

### Time-kill assays.

**FIG 3:**
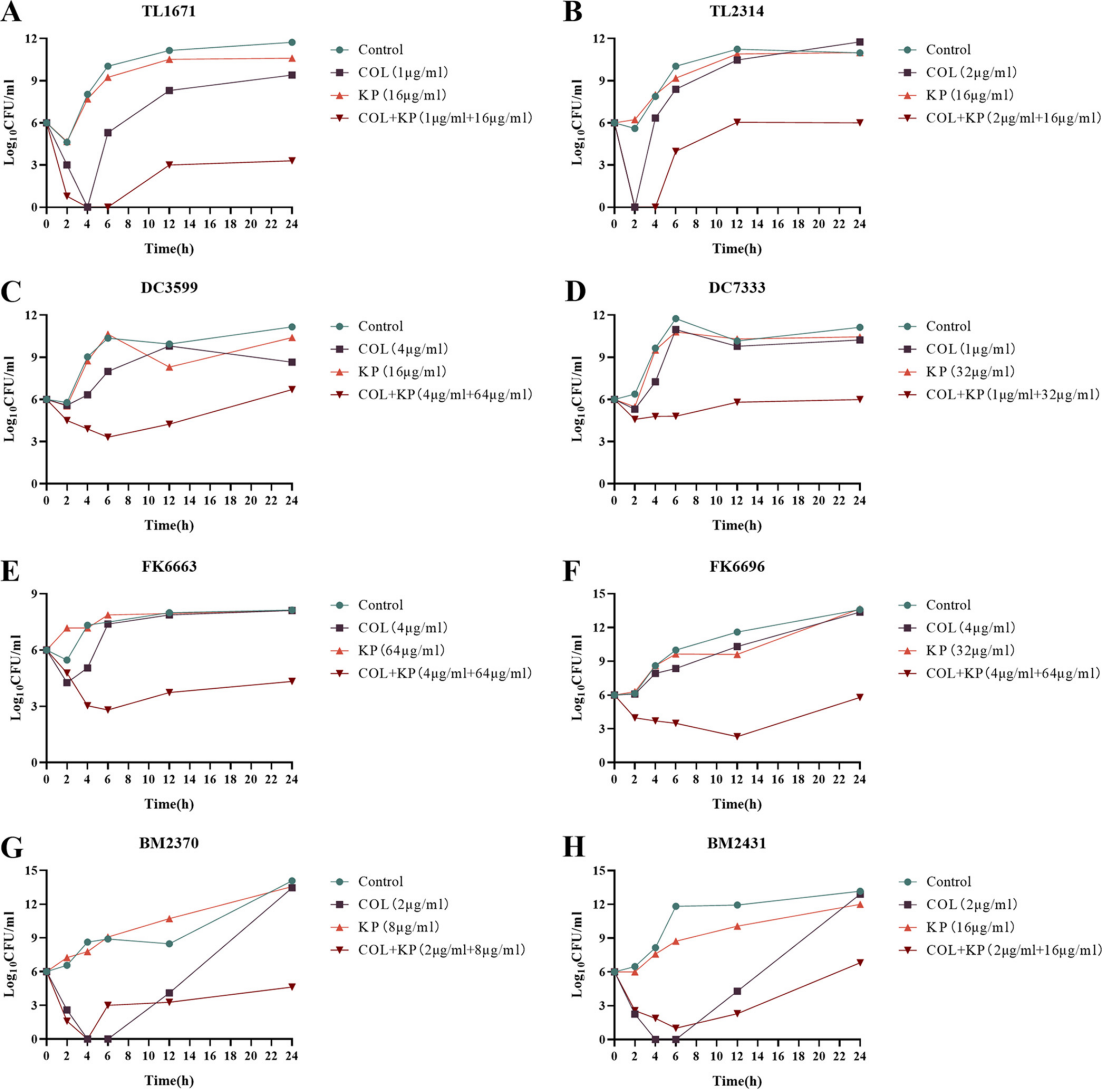
Time-kill curves of COL and KP alone or in combination against Col-R GNB. (A and B) Col-R P. aeruginosa; (C and D) Col-R E. coli; (E and F) Col-R *K. pneumonia*; (G and H) Col-R A. baumannii. All data are reported as the mean ± SD (*n* = 3 per group) from two independent trials.

Time-kill assays further confirmed the synergistic effect of KP and COL on Col-R GNB strains. Time-kill curves against eight Col-R strains (two Escherichia coli; two Pseudomonas aeruginosa; two Klebsiella pneumoniae; and two Acinetobacter baumannii) are revealed in Fig. 3. For these two A. baumannii strains (BM2370, BM2431), the bactericidal effect of COL alone was better than that of the combination within 6 h. However, the cells in the COL alone treated group showed almost linear growth after 6 h. And the bactericidal effect of the combined treatment group was significantly better. As a result, no matter the strain, there were 2 to 6 log_10_ CFU/mL lower in bacterial suspensions treated with COL and KP in combination than in the comparison group.

### Images of scanning electron microscopy.

**FIG 4:**
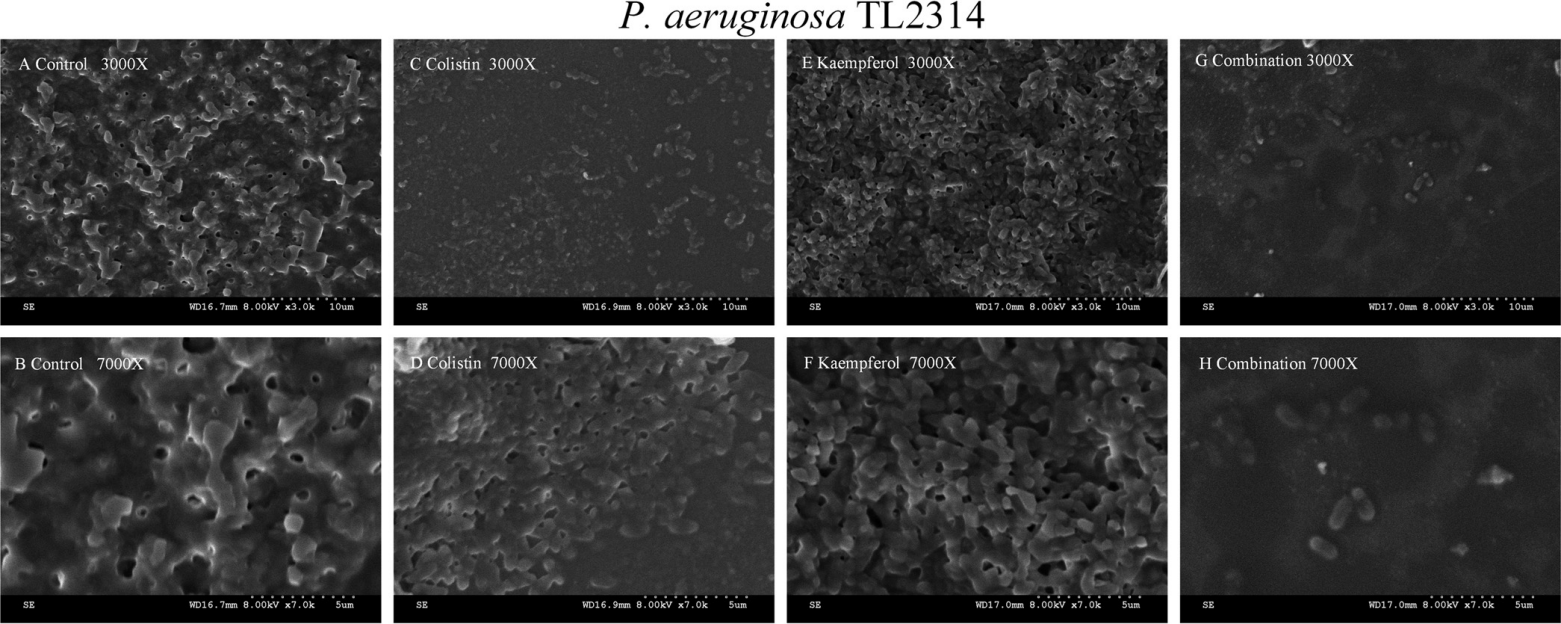
SEM images showing the effect of different treatments on the number and biofilm formation of Col-R P. aeruginosa TL2314. (A) LB broth control at ×3,000 magnification; (B) LB broth control at ×7,000 magnification; (C) COL alone at ×3,000 magnification; (D) COL alone at ×7,000 magnification; (E) KP alone at ×3,000 magnification; (F) KP alone at ×7,000 magnification; (G) COL/KP combination at ×3,000 magnification; (H) COL/KP combination at ×7,000 magnification.

Scanning electron microscopy (SEM) was used to further observe the antibiofilm effect of COL combined with KP. SEM showed that the untreated P. aeruginosa cells had thick biofilms and regular cell morphology. Bacteria treated with COL or KP alone also formed abundant biofilms. In contrast, COL/KP combination treatment demonstrated a considerable drop in bacterium in the treated samples (Fig. 4). Based on these results, we inferred that KP combined with COL has a promising effect on the cells in biofilms.

### *In vivo* treatment verification.

**FIG 5:**
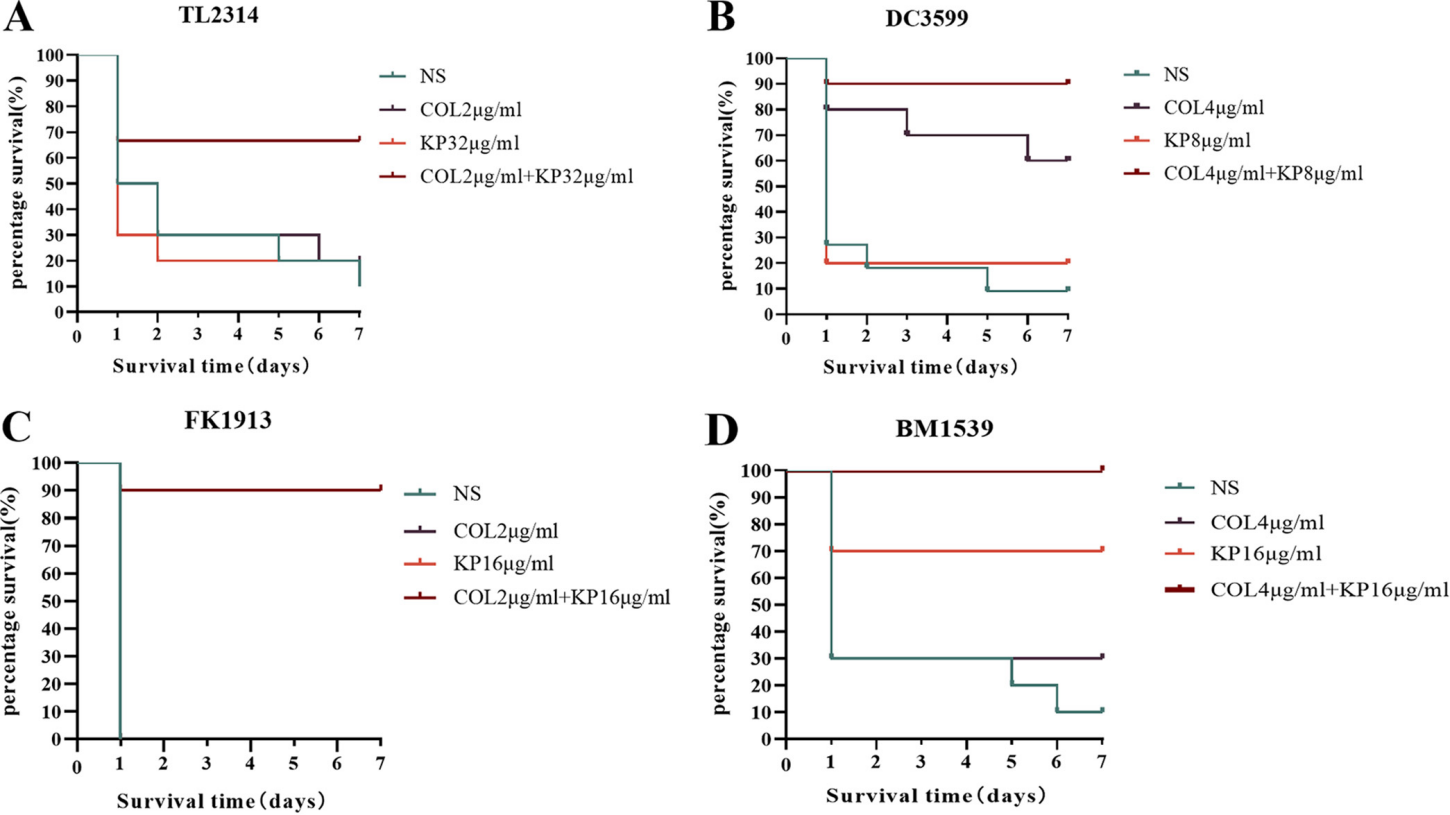
Survival rate of G. mellonella of Col-R P. aeruginosa TL2314, E. coli DC3599, K. pneumoniae FK1913, and A. baumannii BM1539 after 7 days of monotherapy or combination treatment. In Fig. 5C, the survival curves of the NS, COL (2 μg/mL), and KP (16 μg/mL) experimental groups overlap with each other. Except for the COL (2 μg/mL) + KP (16 μg/mL) group, the survival rate of G. mellonella larvae in these experimental groups on the first day was 0. (A) Col-R P. aeruginosa TL2314; (B) Col-R E. coli DC3599; (C) Col-R *K. pneumonia* FK1913; (D) Col-R A. baumannii BM1539.

Now that we have demonstrated the synergistic bactericidal effect of COL and KP *in vitro*, in order to determine the *in vivo* therapeutic effect of the COL/KP combination against Col-R GNB strains, a G. mellonella survival assay was carried out, as shown in Fig. 5. The combination of COL and KP was more effective than the single use. The findings imply that the two medications combined may have beneficial synergistic therapeutic effects on Col-R GNB infections *in vivo*.

### Cytotoxicity potential of the antibacterial agents.

**FIG 6:**
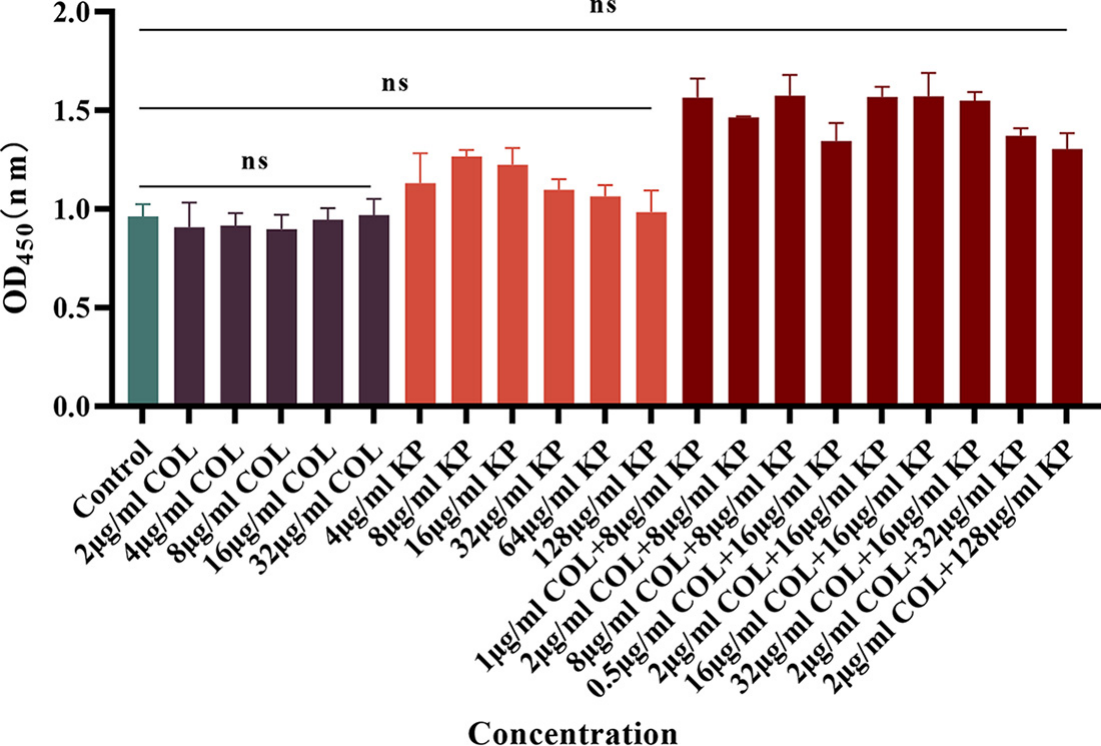
Cytotoxic effects of COL/KP combination with different concentrations under synergistic effects against RAW 264.7 murine macrophage cell line. Data were analyzed by Student's *t* test; (ns, not statistically significant, ****, *P* < 0.0001). OD_450_, optical density at 450 nm.

As shown in Fig. 6, we investigated the potential toxic effects on RAW264.7 cells at the combined concentrations of these two drugs with synergistic effects. The findings demonstrated that at the combined concentrations with synergistic effects, there were no additional toxic effects associated with the combined strategy used in this investigation on RAW264.7 cells.

### Membrane permeability.

**FIG 7:**
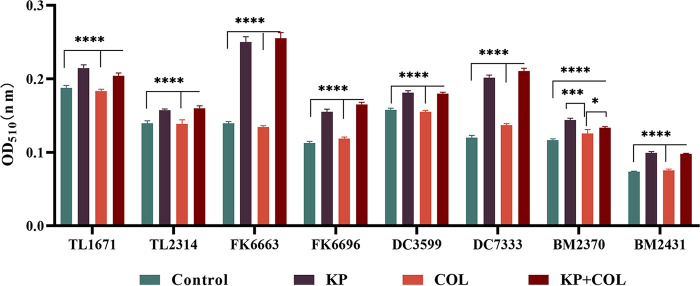
ALP activity in supernatant of bacterial culture with KP and COL alone or in combination. (*, *P* < 0.05; ***, *P* < 0.001; ****, *P* < 0.0001). OD_510_, optical density at 510 nm. A one-way ANOVA was used to compare all KP monotherapy and combination samples to COL monotherapy and the no-treatment control.

We tested the membrane permeability of KP using alkaline phosphatases (ALPs). Compared with the blank group and the COL alone group, the content of ALPs in supernatant of bacterial culture in the KP alone group and the combination treatment group was significantly increased (Fig. 7), indicating that KP could significantly destroy cell membrane integrity and help COL binding more to lipopolysaccharide (LPS) to exert antibacterial activity.

## DISCUSSION

Due to the increasing prevalence of COL resistance and the rising threat of MDR bacteria, it is crucial to find alternative therapeutic options (9). Fruits, vegetables, and herbal remedies are all rich sources of KP, a polyphenol. It is used to treat a variety of illnesses, with a focus on KP's anti-inflammatory properties. Given that their combination has a synergistic effect that lowers the MICs of these medications and lowers the dose required for therapeutic success, the combination of the flavonoid with the aminoglycosides may be an option available to increase the anticipated results in the therapy of Staphylococcus aureus and E. coli (10). KP has been shown to have synergistic activity against P. aeruginosa, E. coli, and S. aureus when combined with other antibiotics such as amikacin and gentamicin, according to research (10). Whereas the antibacterial effect of KP in combination with COL has never been reported. We have combined KP with COL, performed *in vitro* experiments, and designed *in vivo* infection models of G. mellonella to develop a new treatment for Col-R GNB pathogens.

Resistance to antibiotics is rapidly and extensively evolving, especially among Gram-negative bacteria (11). By generating extracellular polymeric molecules, the biofilm condition increases the resistance of the persistent microbes to antimicrobial agents (12). The emergence of traditional antibiotics’ resistance has limited their effectiveness (12). In this work, the synergistic antibacterial and antibiofilm effects of the combination application of COL and KP were investigated. As shown in Table 1, in comparison to COL monotherapy, all experimental strains displayed a 2- to >256-fold increase in susceptibility when KP was included. The time-kill curves in Fig. 3 also show the synergistic effect of combined applications. These findings indicate that KP and COL combination exhibited beneficial synergistic effects.

In addition, to further investigate the synergistic antibiofilm effect of COL and KP, biofilm matrix and cell morphology were examined by SEM and crystal violet staining method. The results demonstrated that most experimental strains' ability to build biofilms could be successfully inhibited by the combination of COL and KP, except for BM2622. However, there was no obvious effect on biofilm eradication. It has been reported that KP prevented biofilm from forming during the initial attachment phase (13). It is speculated that the reason for this result may be as mentioned above. Furthermore, it was observed under SEM that the COL/KP combination disrupted the biofilm of TL2314 and reduced the density of cellular arrangement of the biofilm. However, whether the coadministration of COL and KP affects the expression of COL-related genes or membrane proteins still deserves further study.

We established an infection model of G. mellonella to further assess the *in vivo* therapeutic efficacy of the combination regimen. The findings demonstrated that the combination of COL/KP considerably enhanced G. mellonella survival. It was reported that normal fibroblasts were not affected by the cytotoxic effects of KP on various cancer cells (14). In this study, we performed cytotoxicity assays to assess the safety of the combined concentrations. The results suggested that there was no cytotoxicity of the two drug combinations at the combined concentrations with synergistic effects (15, 16). Additionally, the cell membrane is harmed by the synergistic effects of KP plus COL. It can be assumed from the results that bacterial membrane lysis may be the mechanism by which the combination exerts bactericidal action.

Currently, the therapy of severe infections caused by K. pneumoniae, such as pneumonia and sepsis, is complicated by rising levels of antibiotic resistance. Prior to bacterial challenge, KP-3-O-glucorhamnoside administration substantially reduced expression of the key inflammatory cytokines PGE_2_, TNF-, IL-1, and IL-6 (17). There have been some reports on the antibacterial mechanism of KP alone. KP glycoside was described by Falco-Silva et al. as a potential inhibitor of the efflux pump in bacteria (8). Whereas the synergistic mechanism of COL and KP can be further explored in the future.

In conclusion, this is the first report of synergistic antibacterial and antibiofilm actions of combined KP and COL against Col-R GNB. The combination mentioned in this work may provide a promising new therapeutic option for infections brought on by Col-R clinical GNB.

## MATERIALS AND METHODS

### Bacterial strains and chemicals.

A total of 24 nonduplicated GNB clinical strains were isolated from the First Affiliated Hospital of Wenzhou Medical University in China, including Col-R P. aeruginosa (*n* = 6), A. baumannii (*n* = 6), E. coli (*n* = 6), and K. pneumoniae (*n* = 6). Matrix-assisted laser desorption/ionization time of flight mass spectrometer (MALDI-TOF/MS; bioMérieux, Lyon, France) performs identification of all isolates. All test strains were frozen in Luria Bertani (LB) broth medium (Sigma-Aldrich, St. Louis, USA) supplemented with 30% vol/vol glycerol at −80°C. KP was purchased from Source Leaf Biotechnology Co., Ltd. (Shanghai, China). COL was purchased from Wenzhou Kangtai Biological Technology Co., Ltd. (Zhejiang, China). Alkaline phosphatase kits were purchased from Solarbio Technology Co., Ltd. (Beijing, China).

### Antimicrobial susceptibility assays.

The MICs of COL and KP in 24 clinically isolated GNB isolates were determined by the broth microdilution in cation-adjusted Mueller-Hinton Broth (CAMHB) (Thermo Fisher Scientific, USA). Briefly, the bacteria that had been cultured overnight were adjusted to 0.5 McFarland standard in sterile normal saline (NS), and then diluted at 1:100 in CAMHB. After that, 100 μL of diluted culture were added to each well in Greiner 96-well flat-bottom microtiter plates, resulting in a final bacterial concentration of 7.5 × 10^5^ CFU/mL. Dilution series for ultimate concentrations ranging from 0 to 128 μg/mL for COL and from 0 to 512 μg/mL for KP. Each well had an ultimate volume of 200 μL. The plates were incubated stationarily for 16 h to 18 h at 37°C. The breakpoint point of antibiotics provided by Clinical and Laboratory Standards Institute (CLSI) 2020 is the foundation for the interpretation of antimicrobial susceptibility assays (18). The breakpoints proposed by CLSI were used for COL (susceptible, ≤2 μg/mL; resistant, ≥4 μg/mL). All tests were performed in triplicate for all strains.

### Checkerboard assays.

By modifying the usual checkerboard assay, checkerboard procedures were used to test the combination's *in vitro* synergistic activity against the Col-R GNB (19). COL is 2-fold serially diluted along the *x* axis, while KP is 2-fold serially diluted along the *y* axis to form a matrix (20). The 96-well plates were then incubated at 37°C for 16 h to 18 h, after which the MIC of each drug and the combination of COL and KP were measured. The FICI verified the combined inhibition activities for all strains. The following formula was used to determine the FICI: FICI = FIC_A_ + FIC_B_ (FIC_A_ = MIC_A combined B_/MIC_A_, FIC_B_ = MIC_A combined B_/MIC_B_) (21). Here, COL was designated as “A” and KP as “B.” FICI >2.0, 1.0 <FICI ≤2.0, 0.5 <FICI ≤1.0, FICI ≤0.5 was expected as antagonistic activity, irrelevant activity, additive activity, and synergistic activity, respectively (20).

### Biofilm formation inhibition assays.

In biofilm inhibition test, 12 Col-R strains (three P. aeruginosa, three E. coli, three A. baumannii, and three K. pneumoniae) were used as assay subjects. The bacteria were cultured for 16 h to 18 h at 37°C. After that, sterile NS was used to adjust the cultures to 0.5 McFarland standard. Then the bacteria suspension was diluted 1:100 in LB broth, and finally assigned to a 96-well plate containing KP and COL alone or in combination. Each well was filled with 100 μL of bacterial suspension as well as drug solution. Wells containing 100 μL bacterial suspension + 100 μL LB broth were served as negative controls. After incubation at 37°C for 20 h to 24 h, the culture medium in the 96-well plate was discarded with a pipette. Subsequently, the planktonic cells were removed by washing with NS for 2 to 3 times, and then dried and fixed. After that, 150 μL of 1% crystal violet (CV) dye (Beijing Solarbio Biotechnology Co., Ltd., China) was added to each well for placing 15 min at 37°C. We washed each well twice with NS. Then, 150 μL of anhydrous ethanol were used to solubilize the bound CV. The absorbance was measured at 595 nm with a microplate reader (Multiskan FC) (2, 20). The experiment was repeated in triplicate.

### Removal of developed biofilms assays.

The experimental strains and culture conditions of removal of developed biofilms assays were the same as those of biofilm formation inhibition assays. The bacterial sample was adjusted to 0.5 McFarland standard with sterile NS before being diluted 1:100 in LB broth. We added 200 μL bacterial suspension to each well and incubated at 37°C for 20 h to 24 h. KP and COL were applied either alone or together to each well after discarding the planktonic cells. The media in the 96-well plate was discarded following 20 h to 24 h of incubation at 37°C. The plate was washed 2 to 3 times before being dried for fixation. Then, we added CV and let the plate stand for 15 min. CV was taken out after staining, and NS was used to wash the wells. Following that, anhydrous ethanol was used to solubilize the bound CV as previously mentioned. Utilizing a microplate reader, the absorbance was measured at 595 nm (2, 20). Data from two separate experiments are presented as the mean ± SD (*n* = 3 per group).

### Time-kill assays.

For combination experiments, bacterial cultures were set up as mentioned. In brief, eight strains were randomly selected to conduct experiments, including Col-R E. coli (*n* = 2), P. aeruginosa (*n* = 2), K. pneumoniae (*n* = 2), and A. baumannii (*n* = 2). Bacteria were exposed to KP and COL individually or in combination at a concentration of 1 × 10^6^ CFU/mL. The control group of bacteria was those in the LB medium without antibiotic treatment. All bacterial samples were incubated with moderate shaking at 37°C. At 0 h, 2 h, 4 h, 6 h, 12 h, and 24 h following antibiotic treatment, 100 μL aliquots of the culture (in triplicate) were collected and plated on LB agar. After plates were incubated for 16 h to 18 h at 37°C, bacterial colonies were enumerated. Bactericidal effect was determined as ≥3 log_10_ reduction in CFU/mL at 24 h, and synergistic activity as ≥2 log_10_ reduction by comparing the two medication combinations compared to either drug alone (2, 22).

### Scanning electron microscope.

The strain TL2314 cultured overnight was adjusted to 0.5 McFarland standard with sterile NS. The combination group, KP monotherapy group, COL monotherapy group, and control group were set up. A sterile round glass slide was placed in each group and 100 μL bacterial suspension was added, respectively. To each group, LB broth was added with a ultimate concentration of 1 μg/mL COL or 16 μg/mL KP (2 mL). The culture was left standing at 37°C for 18 h to 24 h. The glass slides were then removed, thoroughly washed using distilled water a total of three times, and fixed overnight with 2.5% (vol/vol) glutaraldehyde. After air drying, the specimens were treated with gold sputtering and observed by SEM (S-3000N, Japan).

### Evaluation of synergistic effects *in vivo* in the G. Mellonella infection model.

The effectiveness of COL alone and in combination with KP was evaluated in the modified survival assays of GNB infected G. mellonella, as described previously (20). The overnight cultures of E. coli (DC3599), P. aeruginosa (TL2314), K. pneumoniae (FK1913), and A. baumannii (BM1539) were adjusted to 1 × 10^5^ CFU/mL with sterile NS. The larvae that received a standard saline injection served as the controls. The bacteria solution (10 μL) was injected into the larva's left hind limb with a microsyringe and treated with the test drug alone or in combination of 7 × MICs after 1 h of infection. The larvae were aerobically incubated at 37°C for 7 days and the survival rates of G. mellonella were noted every 24 h (23). Each experiment was performed 3 times. When larvae did not react to repeated physical stimuli, they were deemed dead.

### *In vitro* cytotoxicity assays.

We evaluated the safety of KP alone and in combination with COL using RAW 264.7 cells, according to previous study (2, 24), with minor modifications. RAW 264.7 cells were purchased from Wuhan Baosai Life Technology Co., LTD., China. RAW 264.7 cells were cultured in Dulbecco's Eagle medium (DMEM) supplemented with 10% heat-inactivated fetal bovine serum (FBS) at 37°C, 5% CO_2_, 95% air, and 100% relative humidity to maintain cell growth until fusion. Then, fused cells were digested with trypsin. Approximately 100 μL cell suspension containing 1 × 10^5^ cells was implanted into each well of the 96-well microplate. After incubation for 24 h, 10 μL of KP (8, 16, 32, 64, and 128 μg/mL) alone and in combination with COL (as shown in Fig. 6) were added to the medium and incubated for 12 h. Wells without drugs were used as negative controls. After incubation, we changed the cell culture medium (25–32). After that, 10 μL of CCK-8 (Dojindo Laboratory, Japan) was added to each well, which was then left to culture at room temperature in the dark for 2 h. Absorbance was recorded at 450 nm with a microplate instrument.

### Outer membrane permeabilization assays.

Col-R P. aeruginosa (*n* = 2), E. coli (*n* = 2), A. baumannii (*n* = 2), and K. pneumoniae (*n* = 2) were inoculated into LB broth at 37°C for 24 h. Then, the bacterial cells (OD_510_ = 0.5) were suspended in PBS containing KP, COL, and KP + COL (the drug concentrations were the same as that in time-kill assays), followed by incubation for 6 h at 37°C. After that, the suspensions were centrifuged for 5 min at 5000 rpm (33). The supernatant were collected and tested for alkaline phosphatase (ALP) activity using the corresponding kits. The absorbance at 510 nm was measured after the water bath for 15 min at 37°C.

### Statistical analysis.

The experiments were all performed in triplicate. The mean ± standard deviation of at least three independent experiments were used to show the data. The statistical analysis was performed by two-sample *t* test, one-way ANOVA and logarithmic grade test (*, *P* < 0.05; **, *P* < 0.005; ***, *P* < 0.001; ****, *P* < 0.0001). All statistically calculated values were carried out with Graph Pad Prism 8.0 statistical software.
