# Supplementary material for: The Antibacterial Activity of Kaempferol Combined with Colistin against Colistin-Resistant Gram-Negative Bacteria
Source: Microbiol Spectr. 2022 Oct 31;10(6):e02265-22. doi: 10.1128/spectrum.02265-22 (PMC9769984; doi:10.1128/spectrum.02265-22)
Supplement: Supplemental file 1 — Tables S1 and S2. Download spectrum.02265-22-s0001.pdf, PDF file, 0.5 MB [file spectrum.02265-22-s0001.pdf]

Table S1 Representative MICs against colistin-resistant GNB.

Species	Strains[a]	Antibiotics[b]	ATM	CAZ	FEP	IPM	CIP	LVX	GEN	TOB	COL	KP
		**Breakpoints (S-R[c])**	8-32	8-32	8-32	2-8	0.5-2	1-4	4-16	4-16	2-4	
	TL1671		8	4	8	2	0.25	1	2	1	**128**	**512**
	**TL1744**		**32**	**32**	16	**16**	**32**	**8**	**≥256**	**32**	**64**	**>512**
P. aeruginosa	TL2314	**MICs (μg/mL)**	16	**32**	16	4	0.5	2	8	2	**32**	**512**
	**TL3008**		4	2	4	**16**	0.5	1	**16**	4	**>128**	**512**
	**TL3086**		**128**	16	16	**≥256**	**16**	**8**	**≥256**	**128**	**>128**	**512**
	**DC3599**		**≥256**	**128**	**≥256**	0.5	**≥256**	**≥256**	**≥256**	**≥256**	**8**	**>512**
	**DC3806**		**64**	**64**	16	1	**4**	**8**	**16**	**16**	**8**	**>512**
	**DC3846**		**128**	**64**	**≥256**	0.5	**≥256**	**128**	**≥256**	**64**	**4**	**>512**
E. coli	**DC4887**	**MICs (μg/mL)**	1	4	**32**	1	**4**	**16**	**16**	8	**16**	**>512**
	**DC5262**		**≥256**	**≥256**	**≥256**	4	2	**16**	**≥256**	**≥256**	**>128**	**>512**
	**DC7333**		**≥256**	**≥256**	**≥256**	**16**	**≥256**	**128**	**128**	**≥256**	**8**	**>512**
	**FK1913**		**≥128**	**≥128**	**≥128**	**32**	**≥128**	**128**	**≥128**	**≥128**	**>128**	**>512**
	**FK3810**		0.0125	**128**	**≥256**	**32**	**≥256**	**128**	**≥256**	**≥256**	8	**>512**
K. pneumoniae	**FK3994**	**MICs (μg/mL)**	**≥256**	**128**	**≥256**	**32**	**≥256**	**64**	**≥256**	**≥256**	**128**	**>512**
	**FK6556**		**64**	**64**	**64**	**16**	**4**	**8**	**16**	**16**	16	**>512**
	**FK6663**		**≥256**	**≥256**	**≥256**	**32**	**≥256**	**≥256**	**≥256**	**≥256**	**128**	**>512**
	**FK6696**		**≥256**	**64**	**≥256**	**128**	**≥256**	**64**	**≥256**	**≥256**	**64**	**>512**
	**BM1539**		16	8	**64**	**16**	**4**	2	1	1	8	**>512**
	**BM1595**		2	**32**	8	4	**64**	**8**	**≥128**	**≥128**	4	**512**
A. baumannii	**BM2370**	**MICs (μg/mL)**	8	**32**	**128**	**8**	**128**	**8**	4	1	4	**512**
	**BM2412**		16	**64**	**64**	**16**	**4**	**8**	4	1	**32**	**512**
	**BM2431**		**64**	**64**	**64**	**16**	**4**	**8**	1	1	4	**512**

[a] Bolded strain number indicates multidrug resistant (MDR) strain.

[b] Bolded values point means resistance.

[c] S-R represents the susceptible (S) breakpoint to resistant (R) breakpoint, according to CLSI supplement M100

(30th edition) and EUCAST.

Abbreviations: GNB, Gram-negative bacteria; ATM, Aztreonam; CAZ, Ceftazidime; FEP, Cefepime; IMP, Imipenem; CIP, Ciprofloxacin; LVX, Levofloxacin; GEN, Gentamicin; TOB, Tobramycin; COL, colistin; KP, kaempferol.

Table S2 Resistance mechanism of colistin in colistin-resistant GNB.

Species	Strains	Resistance mechanism		
P. aeruginosa	TL1671	PmrB (V15I, P216S)		
	TL1722	PmrB (V199I, S257N)	ParR (R146H)	CprS (V181I, R209L)
	TL1736	PmrB (V185A)		
	TL1744	PmrB (V15I, G68S)		
	TL2204	PmrB (Y345H)		
	TL2294	PmrB (G179D, I349V)		
	TL2314		PhoQ (V260G)	
	TL2917	PmrB (G179D)	PhoQ (V260G)	
	TL2967	PmrB (D45E)		
	TL3008	PmrB (A190G)		
	TL3086	PmrB (S27R)		
E. coli	DC3599	*mcr-1*		
	DC3806	*mcr-1*		
	DC3846	*mcr-1*		
	DC4887	*mcr-1*		
	DC5262	*mcr-1*		
	DC7333	*mcr-1*		
K. pneumoniae	FK1913	MgrB (K2E, F28C)		
	FK3810	PmrB (R256G)		
	FK3994	PmrB (R256G)		
	FK6556	PmrB (R256G)		
	FK6663	PmrB (R256G)		
	FK6696	PmrB (R256G)		
A. baumannii	BM1539	LpxA (A182V)	LpxC (P46Q)	
	BM1579	PmrB (A138T)	LpxC (P46Q)	LpxD (A259T)
	BM1595	PmrB (N163I)		
	BM2349	PmrB (N163I)		

	BM2370	PmrB (W11R)		LpxD (N148K)
	BM2412		LpxC (P46Q)	
	BM2431	PmrB (I10T)		
	BM2622	PmrB (A138T)	LpxC (S186R)	LpxD (T289I)
